# Supplementary material for: Decrease in radiative forcing by organic aerosol nucleation, climate, and land use change
Source: Nat Commun. 2019 Jan 24;10:423. doi: 10.1038/s41467-019-08407-7 (PMC6345905; doi:10.1038/s41467-019-08407-7)
Supplement: Supplementary file 1 — Supplementary Information [file 41467_2019_8407_MOESM1_ESM.pdf]

Supplemental Information

Decrease in radiative forcing by organic aerosol nucleation, climate, and land use change

Zhu et al.

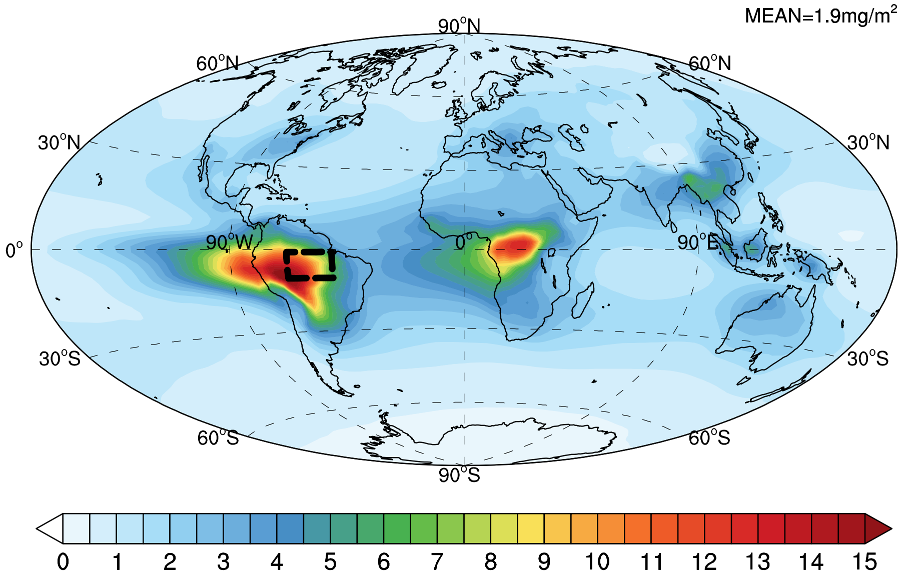

Supplementary Fig. 1 | The burden of total SOA in PD. The dashed box over the Amazon corresponds to the measurement region in ref. [1].

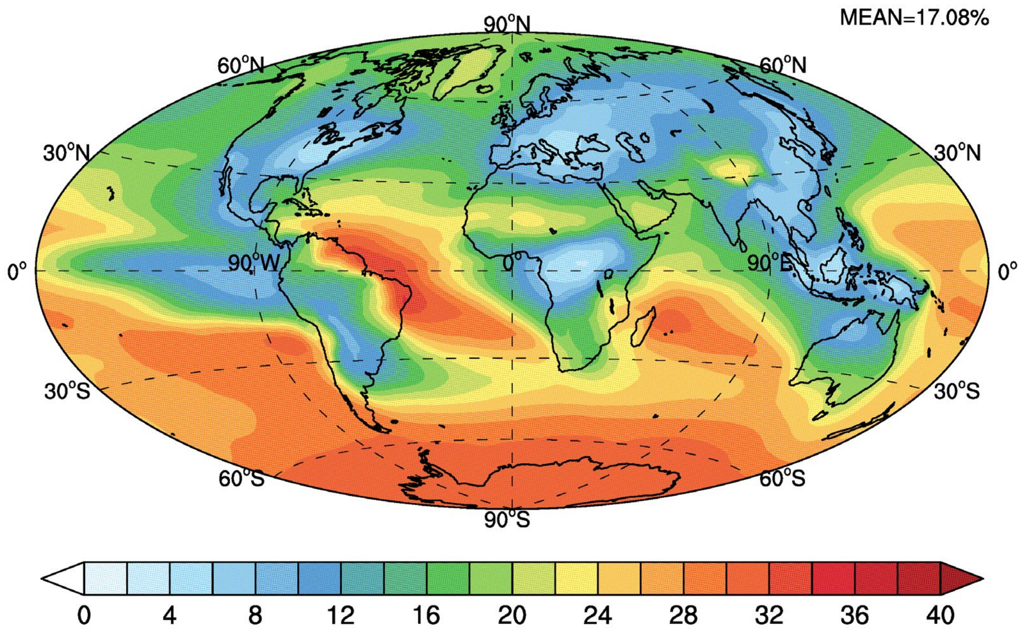

Supplementary Fig. 2 | Vertically integrated burden of newSOA in the PD as a percentage of the total SOA.

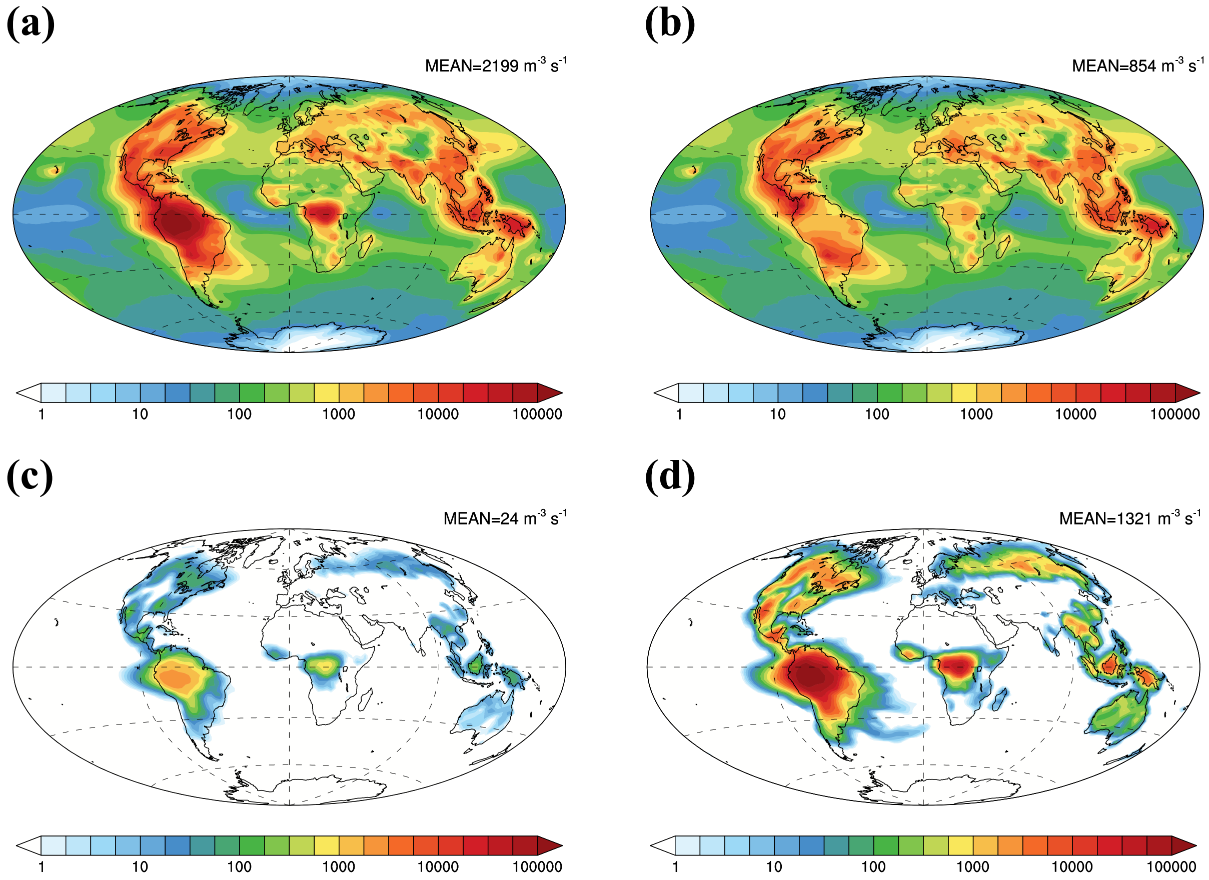

Supplementary Fig. 3 | The vertically averaged total organic nucleation rate (a), heteromolecular nucleation rate of sulfuric acid and organics (b), neutral pure organic nucleation rate (c), and ion-induced pure organic nucleation rate (d) in the PD.

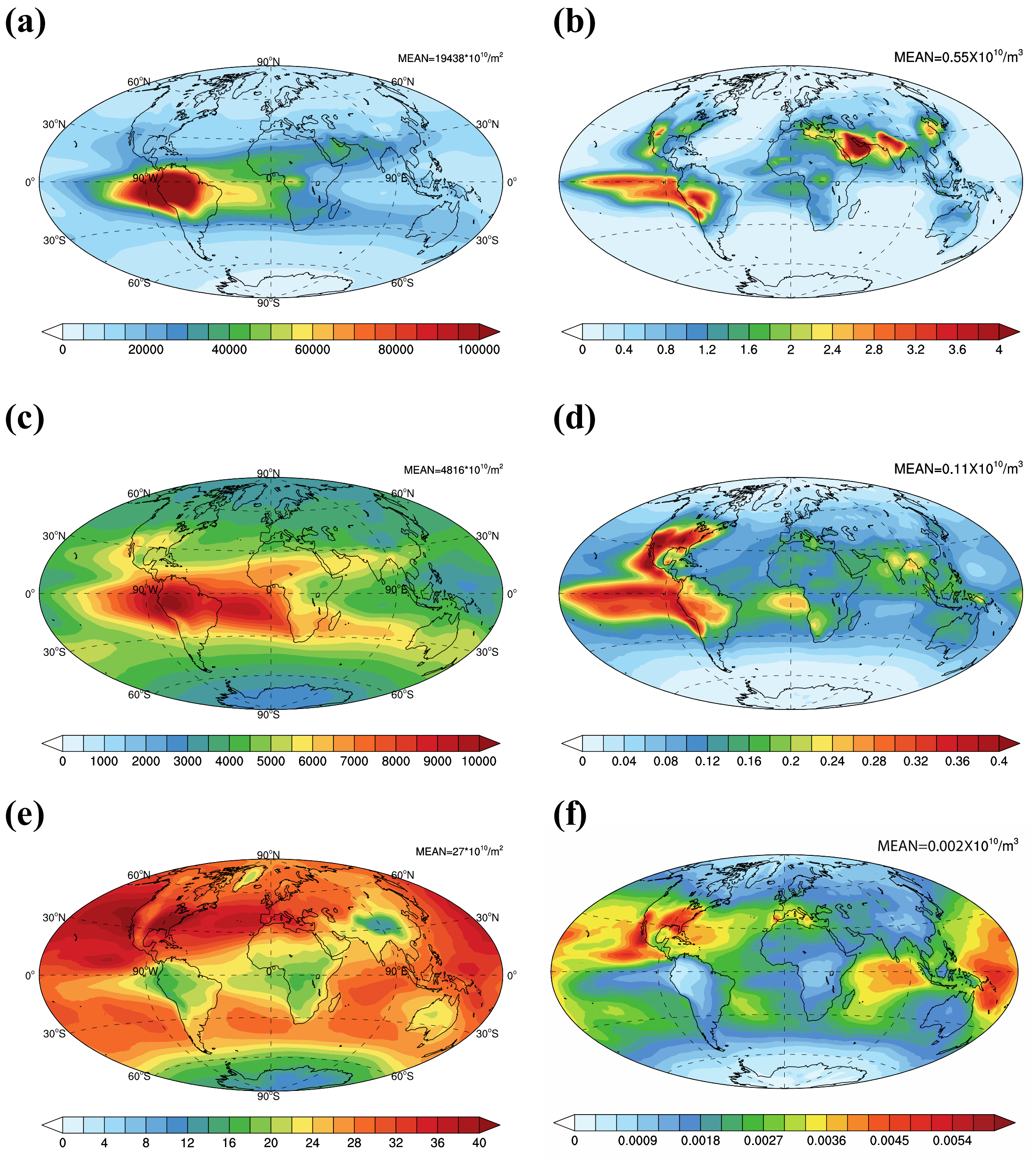

Supplementary Fig. 4 | The column number concentration of newSOA (unit: 10^{10} m^{-2}) in the nucleation mode (a), Aitken mode (c) and accumulation mode (e) in the PD. The number concentration of newSOA in PBL (unit: 10^{10} m^{-3}) in the nucleation mode (b), Aitken mode (d) and accumulation mode (f) in the PD.

(a)

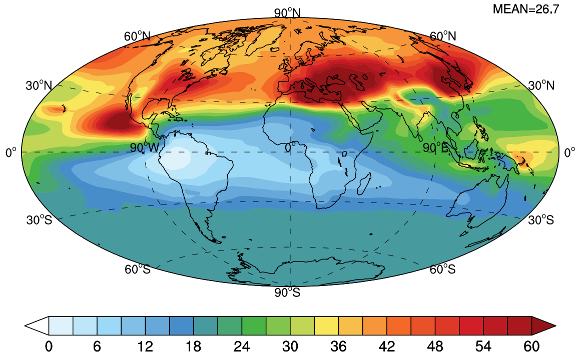

(b)

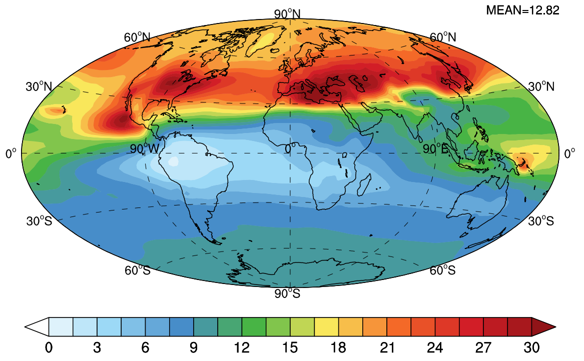

(c)

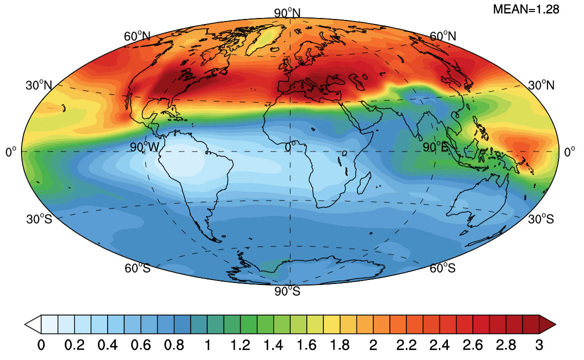

Supplementary Fig. 5 | The mass ratio of vertically averaged sulfuric acid and organics in newSOA in nucleation mode (a), Aitken mode (b) and accumulation mode (c).

(a)

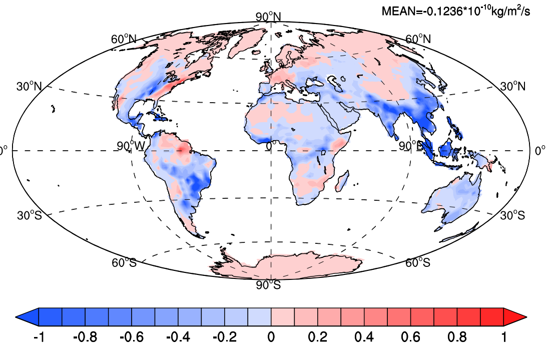

(b)

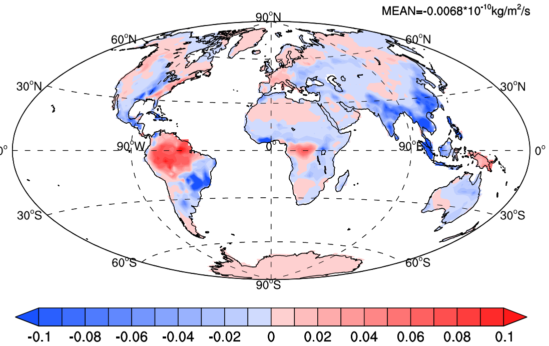

(c)

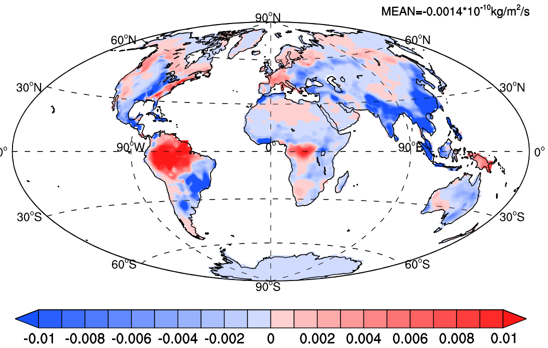

Supplementary Fig. 6 | The difference in the emissions of isoprene (a), α-pinene (b) and limonene (c) between PD and PIall.

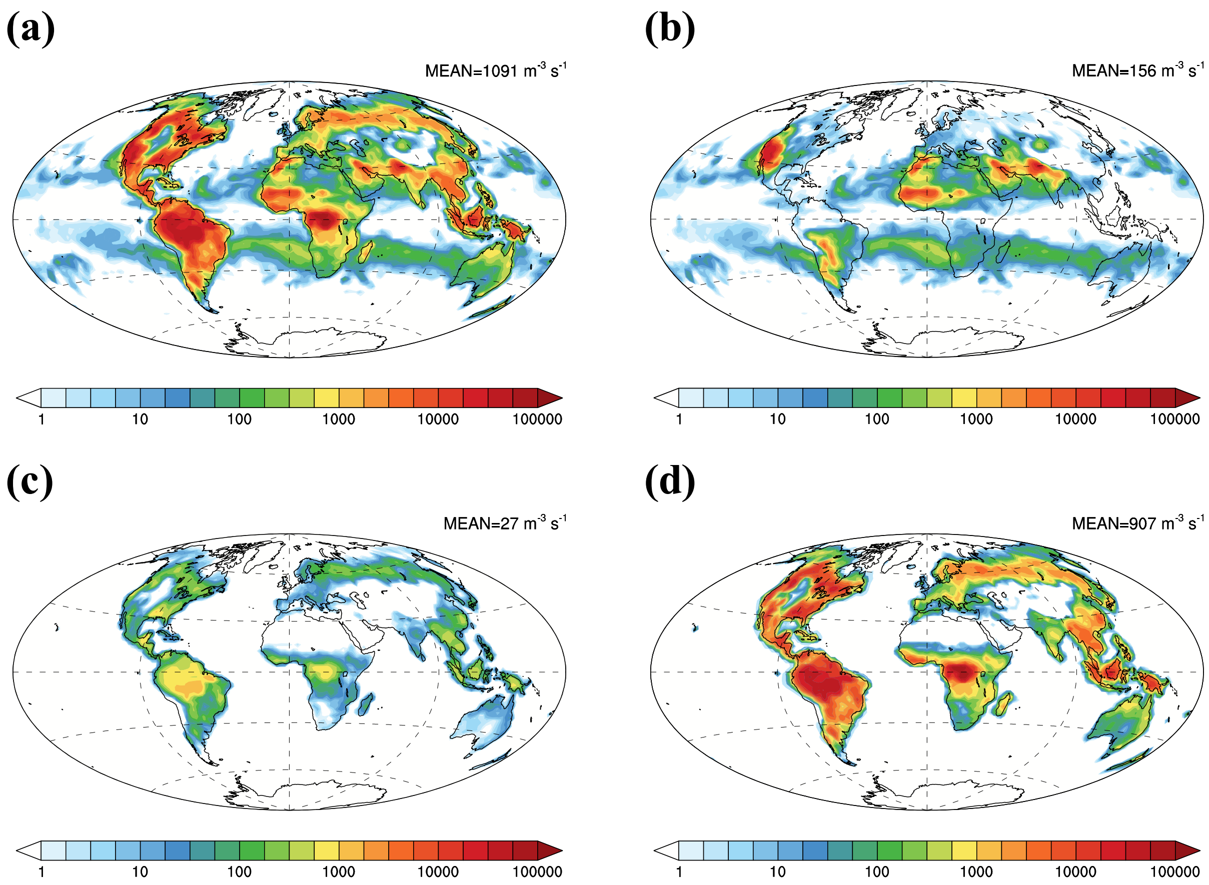

Supplementary Fig. 7 | The vertically averaged total organic nucleation rate (a), heteromolecular nucleation rate of sulfuric acid and organics (b), neutral pure organic nucleation rate (c), and ion-induced pure organic nucleation rate (d) in the PIall.

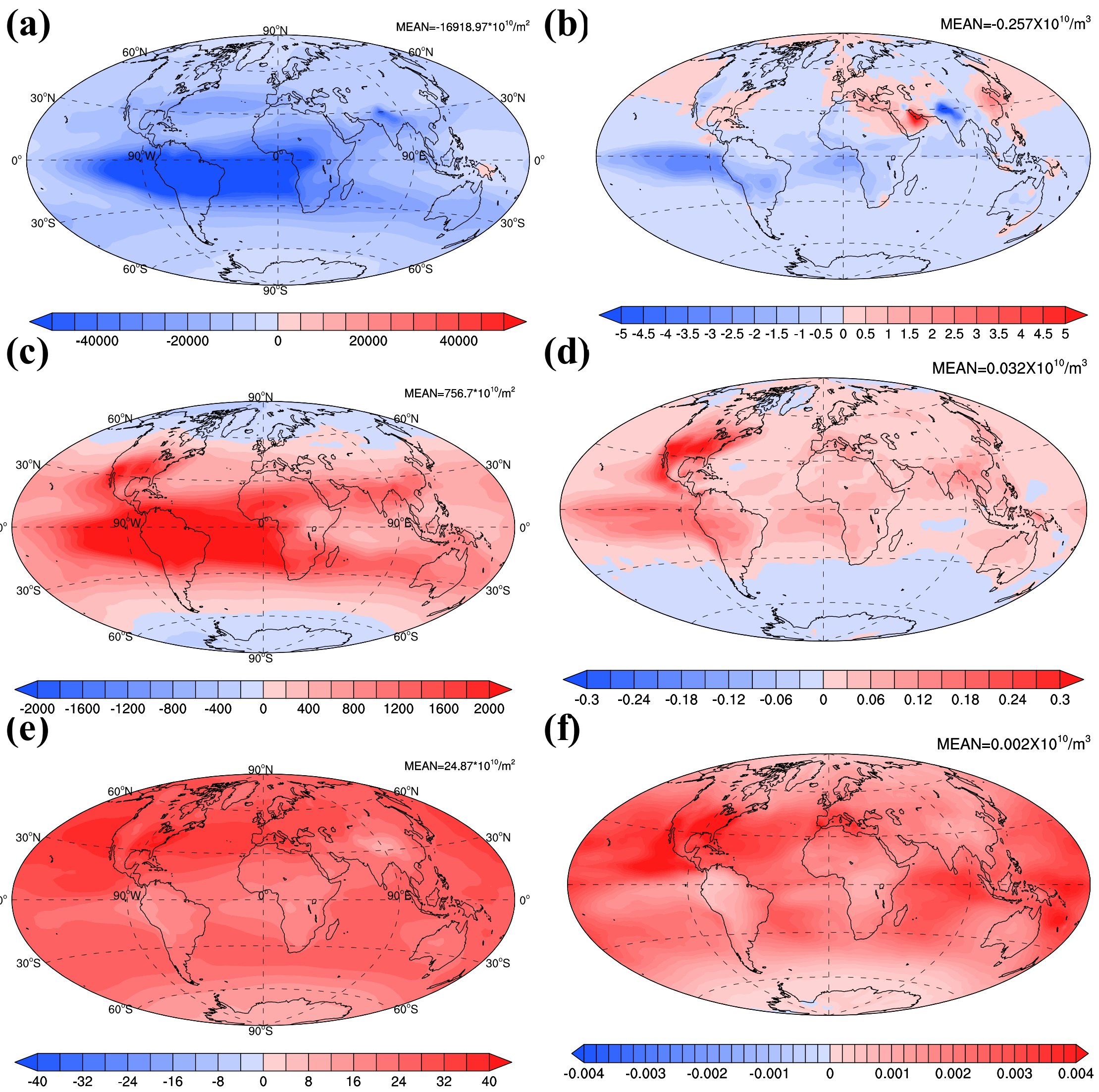

Supplementary Fig. 8 | The difference in the column number concentration of newSOA in nucleation mode (a), Aitken mode (c) and accumulation mode (e) as well as the difference in the number concentration of newSOA in the PBL for the nucleation mode (b), Aitken mode (d) and accumulation mode (f) between PD and PIall.

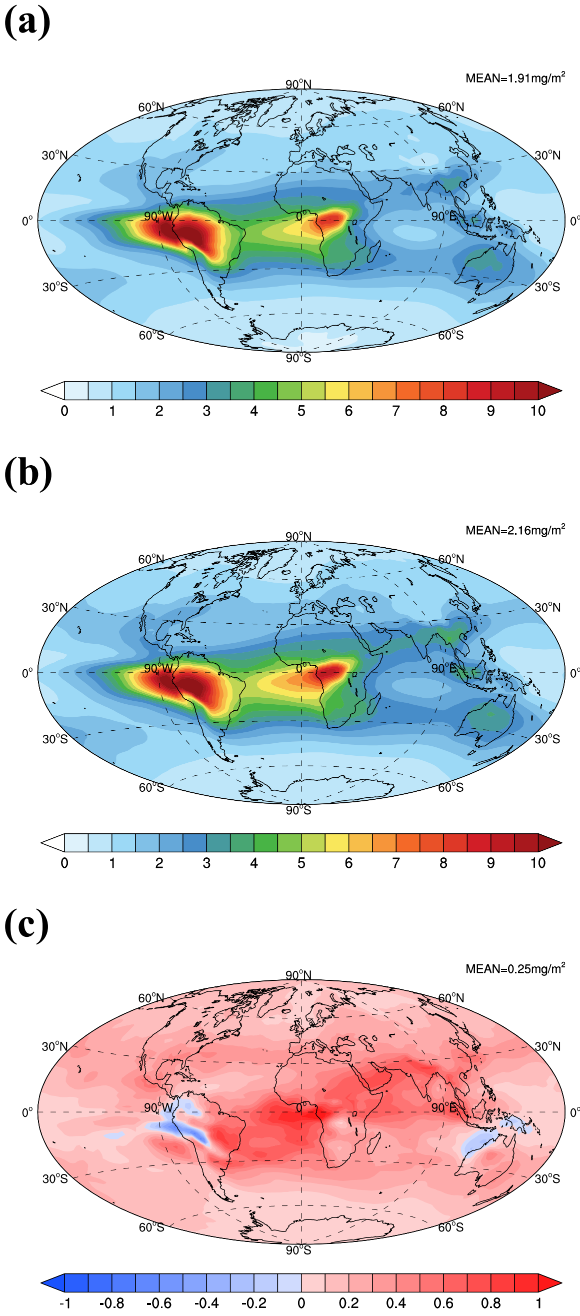

Supplementary Fig. 9 | The burden of total SOA in PIemi (a) and PIall (b) as well as the difference between PIall and PIemi (c).

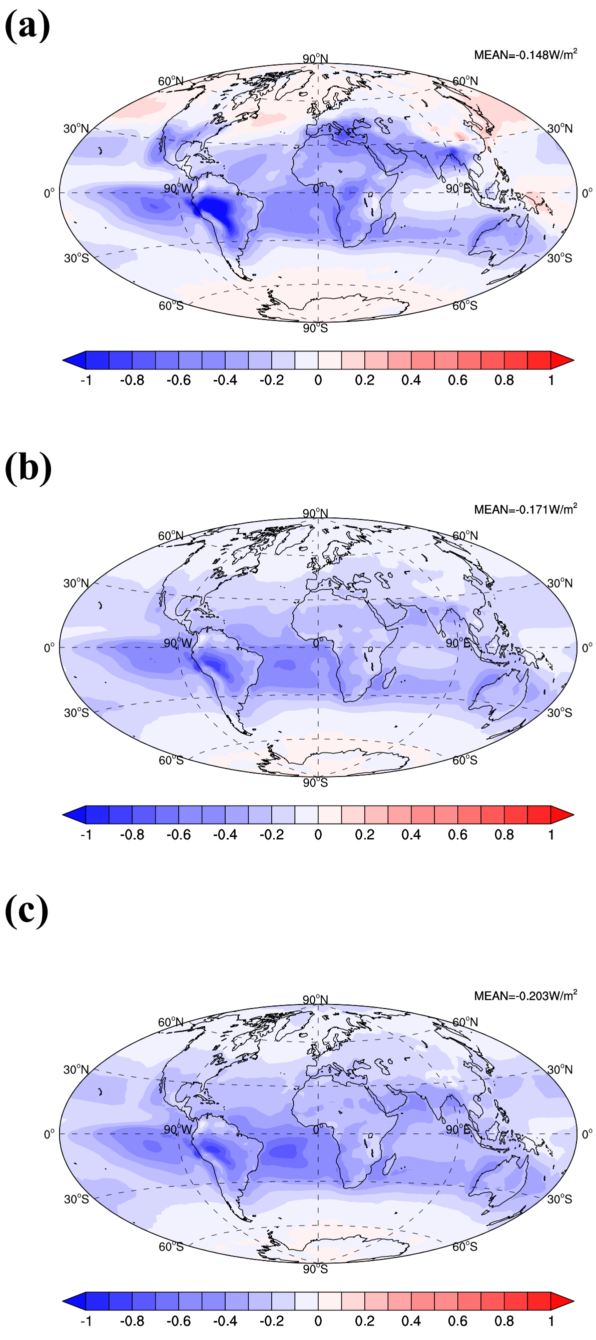

Supplementary Fig. 10 | The DRE of SOA in PD (a), PIemi (b) and PIall (c).

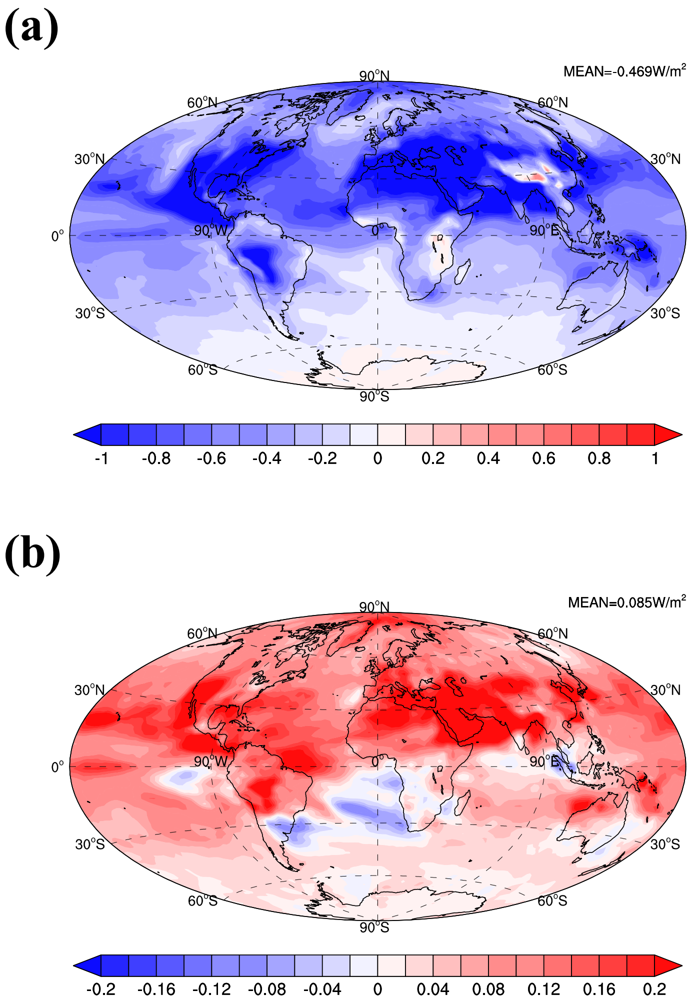

Supplementary Fig. 11 | The DRF of anthropogenic aerosol without organic nucleation with the preindustrial values from PIall (a) and the difference in the DRF of anthropogenic aerosol between with and without organic nucleation (b).

(a)

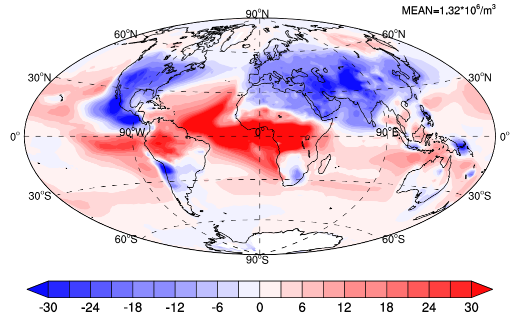

(b)

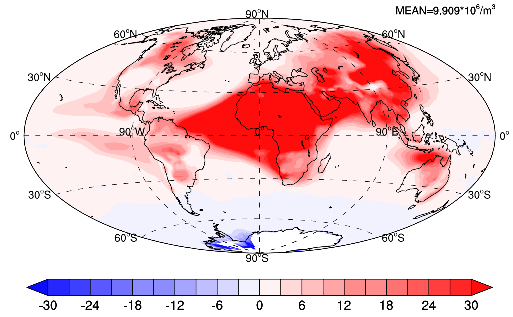

(c)

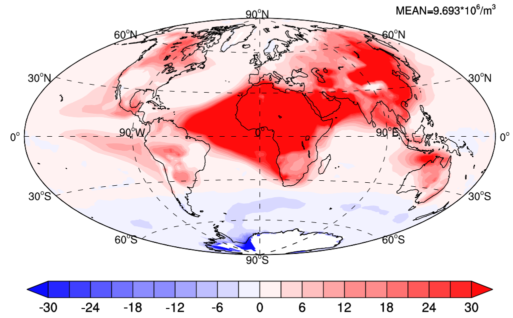

Supplementary Fig. 12 | The change of CDNC at the top of liquid water clouds with and without SOA in the PD (a), PIemi (b) and PIall (c).

(a)

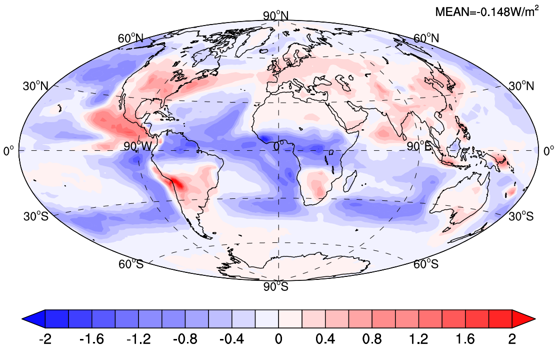

(b)

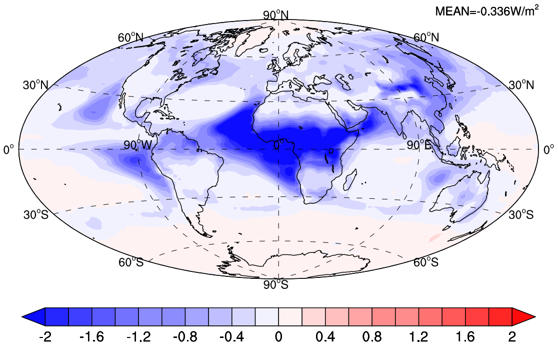

(c)

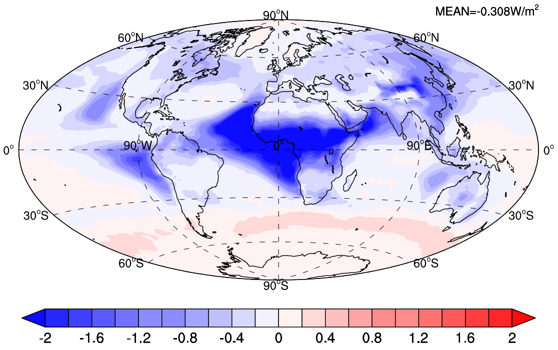

Supplementary Fig. 13 | The AIE due to the addition of SOA in PD (a), PIemi (b) and PIall (c).

(a)

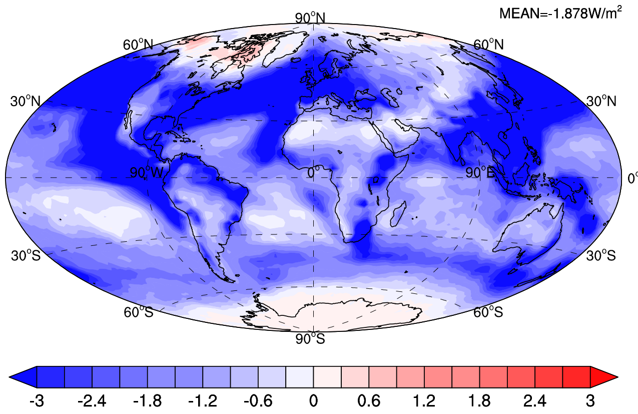

(b)

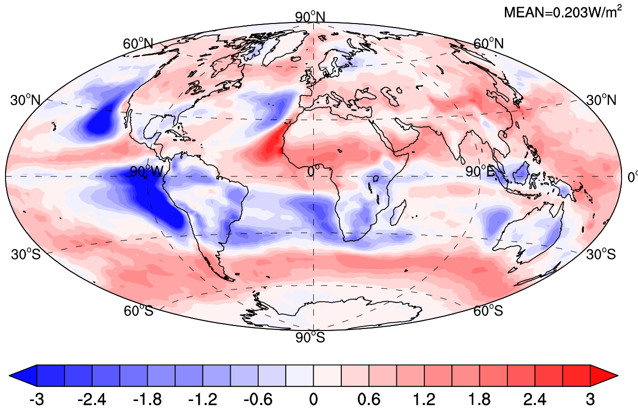

Supplementary Fig. 14 | The IRF of anthropogenic aerosol without organic nucleation with the preindustrial values from PIall (a) and the difference in the IRF of anthropogenic aerosol between with and without organic nucleation (b).

Supplementary Table 1 | The number concentration of newSOA in PBL with organic nucleation (unit: cm^{-3})

	PD	PIemi	PIall
newSOA(nucleation)	5483	6869	8067
newSOA(Aitken)	1067	805	746
newSOA(accumulation)	22	2.8	2.9

Supplementary Table 2 | Summary of the global average aerosol burden and number in PD, PIemi and PIall scheme

		PD	PIemi	PIall
Aerosol Burden (mg m^{-2})	Sulfate (nucleation)	0.001	0.0004	0.0004
	Sulfate (Aitken)	0.22	0.071	0.075
	Sulfate (accumulation)	2.87	0.22	0.20
	Total Sulfate	3.10	0.29	0.28
	Soot (fossil fuel)	0.77	0.16	0.16
	Soot (biomass burning)	1.05	0.94	0.94
	SO4SOA* (nucleation)	0.26	0.11	0.12
	SO4SOA* (Aitken)	1.53	0.39	0.36
	SO4SOA* (accumulation)	0.14	0.015	0.014
Column Aerosol Number (10^{10} m^{-2})	Sulfate (nucleation)	68145	28113	26794
	Sulfate (Aitken)	981	411	473
	Sulfate (accumulation)	154	22	23

* SO4SOA stands for sulfuric acid condensed on newSOA

Supplementary Table 3 | Chemical reactions added to model for simulation of HOMs

No.	Reaction
1	APINENE + O3 => 0.6 × APINOOA + 0.4 APINOOB
2	APINENE + OH => 0.925 PIO2 + 0.075 APINCO2
3	APINOOA => 0.45 × C109O2 + 0.55 × PIO2 + *OH.
4	C10902+RO2=> 0.9 C109O+0.05C109CO+0.05C109OH
5	C109O => 0.8 C89CO3+0.2 DCO3.
6	C89CO3 + HO2 => 0.44 C89CO2 + 0.15 × C89C02H + 0.41 × C89CO3H
7	C89CO3 + RO2 => 0.7 C89CO2 + 0.3 × C89CO2H
8	C89CO2 => 0.8 × C811CO3 + 0.2 × PIO2 (or DCO2)
9	C811CO3 + HO2 => 0.15 PINIC + 0.41 × C811CO3H + 0.44 × PIO2
10	C811CO3 + RO2 => 0.3 PINIC + 0.7 × PIO2
11	2 × C811CO3 => 0.02 DIACYLPER
12	PINIC + OH => PIO2 (or DCO2)
13	DIACYLPER => 0.5 × ESTER
14	DIACYLPER => 0.5 × PINIC
15	APINOOB =>0.5 APINBOO+0.5 PIO2 (or DIO2)
16	APINBOO+H2O=> 0.875 PINAL + 0.125 PINONIC
17	APINBOO+CO=>PINAL
18	APINBOO+NO=>PINAL+NO2
19	APINBOO+SO2=>PINAL+SO3
20	C109O2+HO2=>C109OOH
21	C109O2+NO=>C109O+NO2.
22	C109O2+NO3=>C109O+NO2
23	C109OOH+OH=>C109CO
24	C109CO+OH=>C89CO3
25	C109OOH+hν=> C89CO3
26	C89CO3+NO=>C89CO2+NO2
27	C89CO3+NO3=>C89CO2+NO2
28	C89CO3+NO2=>C89PAN
29	C89PAN=>C89CO3.
30	C89CO3+HO2=> 0.44 C89CO2 + 0.15 C89CO2H+0.41 C89CO3H +0.44 OH+0.15 O3
31	C89CO3H+OH=>C89CO3
32	C89CO2H+OH=>C89CO2
33	C811CO3+NO=>PIO2 + NO2
34	C811CO3+NO3=>PIO2 + NO2
35	C811CO3+NO2=>C811PAN
36	C811PAN=>C811CO3
37	C811CO3+HO2=>0.41 C811CO3H + 0.44 PIO2 + 0.15 PINIC
38	C811CO3H+OH=>C811CO3
39	APINCO2+RO2=>0.7 APINCO + 0.3 APINCOH
40	APINCOH+OH=>APINCO

*No. 1-14 reactions refer to ref. [2] and No. 15-40 reactions refer to Master Chemical Mechanism (MCM). DIACYLPER, PINIC, APINCOH and APINCO are regard as HOMs during nucleation.

S1. Further model evaluation by comparison with observations

Here, we examine the capability of the model to reproduce observations outside of the Amazon. We compare the annual average surface concentration of total organic carbon (OC) with observations in the 28 regions defined by the 196 sites of Interagency Monitoring of Protected Visual Environments (IMPROVE) network in United States during 2005-2008[3] (Table S4) and the 24 sites of the European Monitoring and Evaluation Program (EMEP) network where data are available from 2000 to 2010[4] (Table S5). The model underestimates the OC concentration in most regions compared to observations with an average normalized mean bias (NMB) of -28% for the IMPROVE network and -67% for the EMEP network, but generally is within a factor of 2 of the observations at most sites. Due to the coarse resolution used in our model, local high levels of OC are not able to be predicted. One reason for the underestimation at EMEP sites is that some large particles observed in EMEP sites are not captured in our model since all organics are treated as submicron particles while 70% of the EMEP sites only report PM_{10}. Moreover, the measurements at most of EMEP sites show a high concentration of OC in winter which probably has a source associated with primary emissions from biomass burning [5]. Domestic combustion in winter is not fully represented in our emission database and the emission of POC in the model does not have any significant monthly variation due to the lack data to support a temporal variation. As a result,

our model fails to reproduce the high level of OC concentrations from primary emissions in the winter. The model is able to capture the spatial pattern of OC concentrations in the United States with a spatial correlation coefficient of 0.70 and in Europe with a spatial correlation coefficient of 0.43.

The vertical profiles of OC concentrations predicted by the model are compared with 17 aircraft campaigns from 2001 to 2009 over the world (Figure S15). Details of these campaigns was synthesized in ref. [6]. The model is able to reproduce the profile of OC concentration in the campaigns conducted in most of the polluted regions (ACE-Asia, EUCAARI, ADIENT, ADRIEX, TexAQS) and remote regions (OP3, VOCALS-UK, ITOP, TROMPEX). The model always underestimates the OC concentration for campaigns conducted in the fire-influenced regions (DODO, DABEX, AMMA, ARCTAS) due, perhaps, to complex features of the emission sources such as temporally varying biomass burning emissions which are not represented in our model. The model always shows a decrease of the OC concentration with an increase in altitude, but fails to capture some of the peaks observed in the campaigns which are caused by occasional plume.

Organic nucleation makes a large contribution to the aerosol number concentration. In addition to the comparison of aerosol number concentration in the Amazon in the main text, we evaluate the model ability to simulate surface aerosol number concentrations at 27 sites over the world (Table S6). The NMB of aerosol

number concentration between the simulation and observations are less than 20% at more than 40% of the sites, but the model also overestimates the aerosol number concentration by a factor of >2 at four sites. In total, the model overestimates the aerosol number concentration with an NMB of 19% over the 27 sites. There are 7 sites at the top of mountains with elevations higher than 2000 m. Due to the coarse resolution of the model, it is hard to reproduce the surface concentration at the top of high mountains. Without these mountain sites, the model only overestimates the aerosol number concentration by 9.7%. Moreover, the model is able to capture the spatial distribution of the aerosol number concentration with a spatial correlation coefficient of 0.85. The three sites in Finland (i.e. Varrio, Hyytiälä and Pallas) and the Whistler Mountain site in Canada are expected to strongly influenced by BVOCs and organic nucleation. The correlation coefficient between monthly simulated number concentration of newSOA and observed aerosol number concentration at these sites are all higher than 0.6, which indicates the model is able to reproduce the seasonal variation pattern of aerosol number concentration in regions with organic nucleation.

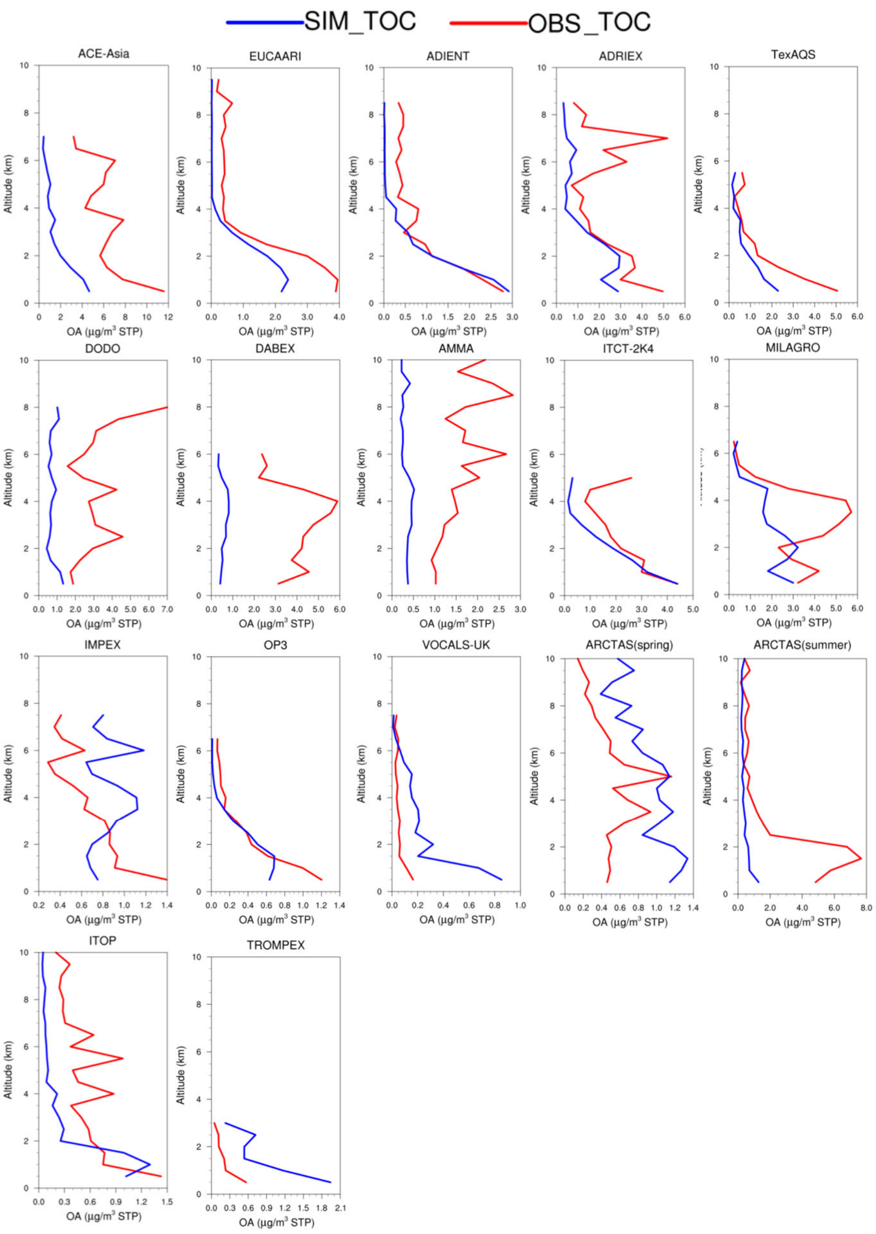

Supplementary Fig. 15 | The mean vertical profile of the observed (red) and simulated (blue) concentration of organic aerosol for 17 field campaigns[6].

Supplementary Table 4. Comparison of the modeled OC concentration with observations from the 28 regions of the IMPROVE network

NO.	Region	Observation (μg C/m3)	Simulation (μg C/m3)	NMB
1	Alaska	0.364	0.090	-75%
2	Appalachia	1.569	1.910	22%
3	Boundary Waters	0.907	0.582	-36%
4	California Coast	0.869	0.833	-4%
5	Central Great Plains	1.171	0.762	-35%
6	Central Rockies	0.532	0.347	-35%
7	Colorado Plateau	0.611	0.375	-39%
8	Columbia River Gorge	1.269	0.503	-60%
9	Death Valley	0.715	0.415	-42%
10	East Coast	1.345	1.019	-24%
11	Great Basin	0.645	0.297	-54%
12	Hawaii	0.145	0.154	6%
13	Hells Canyon	1.232	0.343	-72%
14	Mid South	1.475	1.013	-31%
15	Mogollon Plateau	0.807	0.484	-40%
16	Northeast	1.049	1.231	17%
17	Northern Great Plains	0.816	0.306	-62%
18	Northern Rockies	1.115	0.345	-69%
19	Northwest	0.741	0.522	-30%
20	Ohio River Valley	1.689	1.610	-5%
21	Oregon/N.California	1.118	0.596	-47%
22	Sierra Nevada	1.278	0.834	-35%
23	Southeast	1.785	0.890	-50%
24	S. Arizona	0.703	0.567	-19%
25	S. California	0.939	1.139	21%
26	Virgin Islands	0.123	0.059	-53%
27	West Texas	0.687	0.462	-33%
28	Ontario	1.119	1.396	25%

NMB: normalized mean bias

Supplementary Table 5. Comparison of the modeled OC concentration with observations at the EMEP network sites

NO.	Region	Observation (μg C/m^3)	Simulation (μg C/m^3)	NMB
1	Illmitz	4.69	2.40	-49%
2	Payerne	2.32	1.10	-53%
3	Rigi	1.00	0.70	-30%
4	Kosetice	3.56	1.28	-64%
5	Waldhof	4.34	1.06	-75%
6	Melpitz	2.90	1.14	-61%
7	Campisabalos	2.15	0.47	-78%
8	Montseny	2.03	0.68	-67%
9	Virolahti II	2.12	1.41	-34%
10	Puy de Dome	0.97	0.45	-53%
11	Harwell	1.94	0.61	-68%
12	Edingburgh	1.51	0.30	-80%
13	Mace Head	1.31	0.13	-90%
14	Ispra	9.03	1.10	-88%
15	Belogna	6.03	1.01	-83%
16	Kollumerwaard	2.44	0.66	-73%
17	Birkennes	0.96	0.32	-67%
18	Birkenne II	0.90	0.32	-65%
19	Diabla Gora	1.64	1.73	5%
20	Braganca	4.09	0.58	-86%
21	Vavihill	1.62	0.82	-49%
22	Aspvreten	1.85	1.05	-43%
23	Iskrba	3.38	1.59	-53%

NMB: normalized mean bias

Supplementary Table 6. Comparison of observed and simulated aerosol number concentration (Unit: 10^{10} m^{-3}) at various sites throughout the world

No.	Site	Observation	Simulation	NMB
1	Whistler Mountain	672	1740	159%
2	Alert	207	112	-46%
3	Jungfraujoch	509	2011	295%
4	Hohenpeissenberg	3613	3443	-5%
5	Neumayer	433	375	-13%
6	Izana	2835	1707	-40%
7	Varrio	898	873	-3%
8	Hyytiala	2209	1898	-14%
9	Pallas	770	1089	41%
10	Puy de Dome	2813	2466	-12%
11	Harwell	5044	5164	2%
12	Mace Head	2318	1069	-54%
13	Mt Cimone	1791	2980	66%
14	Preila	2716	2788	3%
15	Zeppelin mountain	337	301	-11%
16	Cape San Juan	697	642	-8%
17	Lulin	1222	1559	28%
18	Barrow	234	558	138%
19	Bondville	4387	8156	86%
20	Mauna Loa	589	640	9%
21	Boone	2969	5173	74%
22	Matatula	214	577	169%
23	Southern Great Plains	4385	3876	-12%
24	South Pole	70	140	100%
25	Trinidad Head	903	1279	42%
26	Steamboat Spring	2059	3370	64%
27	Cape Point	1157	655	-43%

NMB: normalized mean bias

S2. Comparison of nucleation rate with Gordon et al. (2017)[7]

Different models use different schemes to determine the nucleation rate, and this adds significant uncertainty to the predicted aerosol numbers concentration. Ref.[7] developed their model to include pure organic nucleation. We compared the fractions of new particle formation from each pathway within 5.8km (same altitude used for the results reported in ref.[7]) from our simulation with those in ref.[7]. Our model doesn't include the nucleation of sulfuric acid with ammonia, so we compared the nucleation rate of binary sulfuric acid-water nucleation in our model with the sum of "SA-NH3", "SA-NH3-ion" and "SA-ion" in ref.[7]. Also, since our model does not include ion-induced heteromolecular nucleation of sulfuric acid and organics which is included in their model, we compared the heteromolecular nucleation rate in our model with the sum of "SA-org" and "SA-org-ion" in their results. The comparison for the present day is shown in the Table S7. The fraction of new particle formation from sulfuric acid is 10% higher in our model than that from the Gordon et al. model. That is probably because there is a large fraction of organic nucleation that occurs in middle and upper troposphere in our model while organic nucleation mainly occurs near the surface when it is independent of temperature in the Gordon et al. study. The fraction of new particle formation from heteromolecular nucleation of sulfuric acid and organics (HET) is smaller in our model, which may be caused by the neglect of the ion-induced HET pathway. As a result, more HOMs would take part in the ION pathway to form new organic particles in our model than in the Gordon et al. study, leading to a higher fraction of new particle formation from ION in our result.

Supplementary Table 7 The fraction of new particle formation from each pathway in our study and Gorden et al. (2017)[7]

This study		Gordon et al. (2017)	
Pathway	Fraction	Pathway	Fraction
ION	23.2%	org-ion	4.1%
NON	0.6%	Neutral organic	0.4%
HET	17.8%	SA-org<br>SA-org-ion	47.0%
H_2SO_4+H_2O	58.4%	SA-ion<br>SA-NH_3<br>SA-NH_3-ion	48.5%

S3. Uncertainty experiments

S3.1 Uncertainty of temperature dependence

The base model in the main text includes new particle formation by organics and has a temperature dependence of exp(-(T-278)/10) based on quantum chemical calculations of cluster binding energies[8]. However, this temperature dependence has not yet been determined by experimental evidence, so the temperature dependence has a significant uncertainty. We used a sensitivity test to examine the radiative forcing without including any temperature dependence for organic nucleation. When the nucleation scheme without temperature dependence is applied in the model, the organic nucleation and the number concentration are decreased by more than 50%, especially in the upper troposphere. When we include the temperature dependence, organic nucleation is suppressed at higher temperatures, whereas without the temperature dependence, there are a large number of newSOA particles formed within the lower troposphere leading to a peak in the aerosol number concentration at around 1.5 km in the Amazon and there is a small peak in the aerosol number concentration at around 12 km, which is about half as large as the peak predicted by the base model in the main text. The DRF of anthropogenic aerosol is estimated to -0.464 W m^{-2} without the temperature dependence while the IRF of anthropogenic

aerosol is -1.706 W m^{-2}. The total RF of anthropogenic aerosol is increased by 5.4% compared to the RF estimated by the base model in the main text.

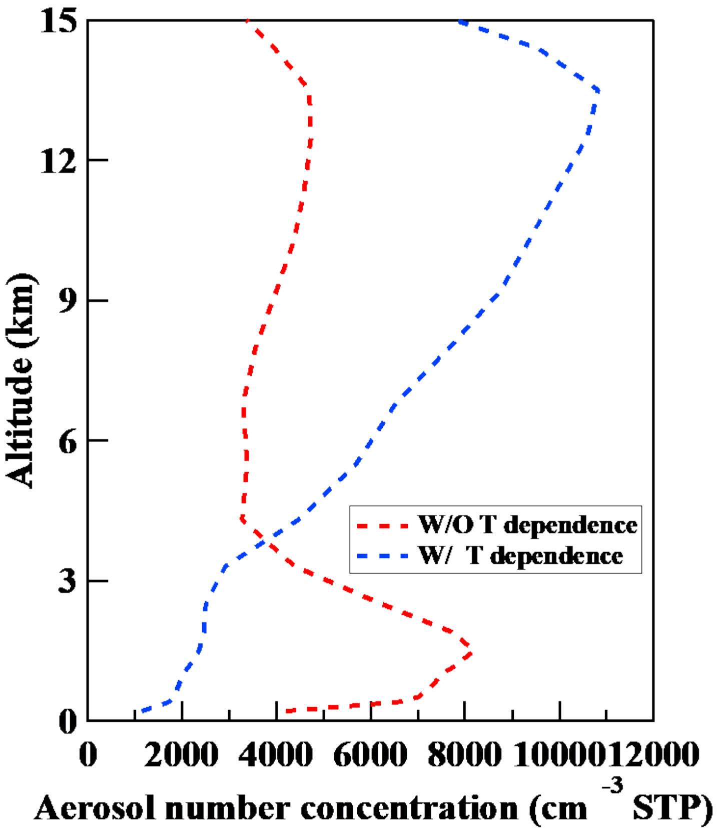

Supplementary Fig. 16 | Multi-year average September and October vertical profile of aerosol number concentration with diameter above 10 nm in the lower troposphere and 20 nm in the upper troposphere over the Amazon in the sensitivity experiment without using the temperature dependence of the nucleation rates (red) and the base experiment (blue).

S3.2 Uncertainty of condensed organics on newSOA

In the base model, the newSOA are grown by sulfuric acid and organics from partitioning of HOMs and SVOC. IEPOX, glyoxal and methylglyoxal are taken up only by new sulfate particles in the base model. However, sulfuric acid is the most

important constituent in newSOA and could provide an acidic and wet environment, so that we assume IEPOX, glyoxal and methylglyoxal are also able to be taken up by newSOA as is done with the new sulfate particles. Therefore, the low-volatility products formed from IEPOX, glyoxal and methylglyoxal may also contribute to the growth of newSOA. In this sensitive experiment, organics make a much larger contribution to the growth of newSOA than that in the base model. The organics become the dominant constituent in the newSOA particles in the tropics, while sulfuric acid is still important for the growth of newSOA in industrial regions. The mass concentration of organics on newSOA in each mode is larger by a factor of 4-7 than that of sulfuric acid in the tropics in the PD (Figure S17). In this sensitivity experiment, the number concentration of newSOA in the Aitken and accumulation modes is increased by 43% and 258% in the PD compared to those predicted by the base model. As a result, the DRF of anthropogenic aerosol is estimated to be -0.463 W m^{-2} and the IRF of anthropogenic aerosol is -1.850 W m^{-2}. The total RF of anthropogenic aerosol is increased by 12% compared to the RF estimated by the base model in the main text.

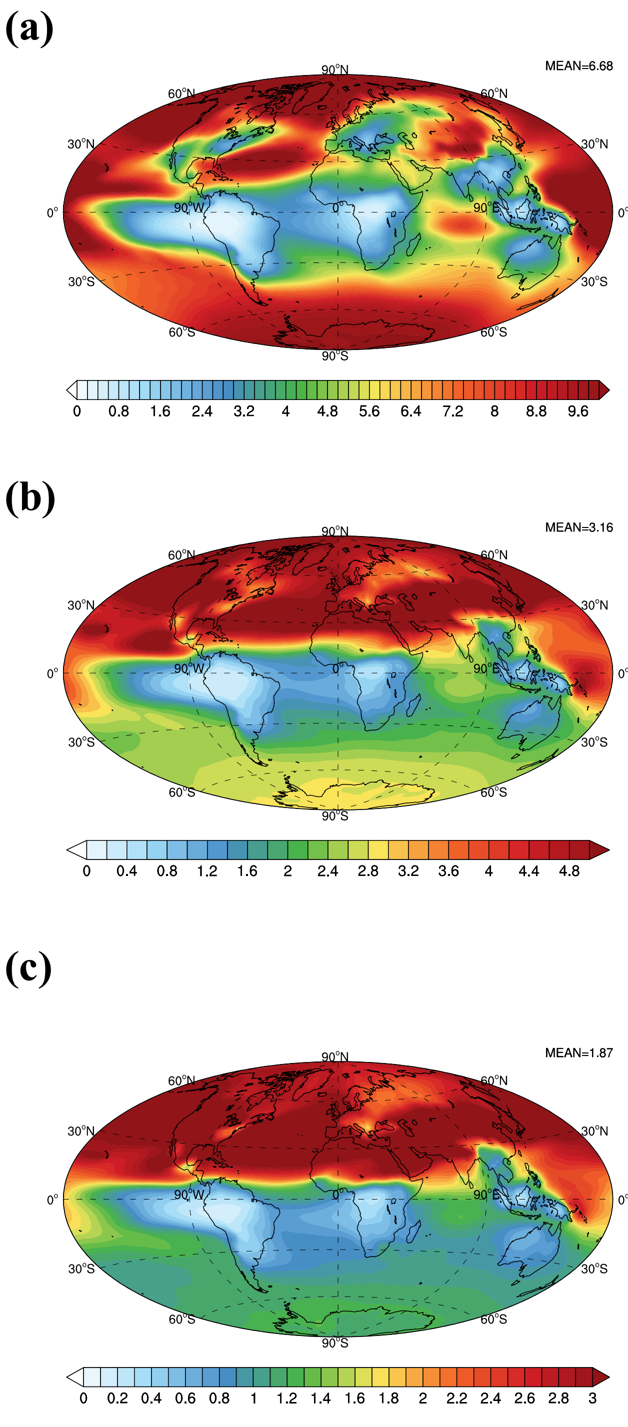

Supplementary Fig. 17 | The mass ratio of vertically averaged sulfuric acid and organics in newSOA in nucleation mode (a), Aitken mode (b) and accumulation mode (c) in the sensitivity experiment.

1 Andreae, M. O. *et al.* Aerosol characteristics and particle production in the upper troposphere over the Amazon Basin. *Atmospheric Chemistry and Physics* **18**, 921-961, doi:10.5194/acp-18-921-2018 (2018).

2 Zhang, X. *et al.* Formation and evolution of molecular products in alpha-pinene secondary organic aerosol. *Proc. Natl. Acad. Sci. U. S. A.* **112**, 14168-14173, doi:10.1073/pnas.1517742112 (2015)

3 Hand, J. *et al.* Spatial and seasonal patterns and temporal variability of haze and its constituents in the United States, Report V. *Colorado State University, Fort Collins CO* (2011).

4 Torseth, K. *et al.* Introduction to the European Monitoring and Evaluation Programme (EMEP) and observed atmospheric composition change during 1972-2009. *Atmospheric Chemistry and Physics* **12**, 5447-5481, doi:10.5194/acp-12-5447-2012 (2012).

5 Gelencser, A. *et al.* Source apportionment of PM2.5 organic aerosol over Europe: Primary/secondary, natural/anthropogenic, and fossil/biogenic origin. *Journal of Geophysical Research-Atmospheres* **112**, 12, doi:10.1029/2006jd008094 (2007).

6 Heald, C. L. *et al.* Exploring the vertical profile of atmospheric organic aerosol: comparing 17 aircraft field campaigns with a global model. *Atmospheric Chemistry and Physics* **11**, 12673-12696, doi:10.5194/acp-11-12673-2011 (2011).

7 Gordon, H. *et al.* Causes and importance of new particle formation in the present-day and preindustrial atmospheres. *Journal of Geophysical Research: Atmospheres* **122**, 8739-8760, doi:10.1002/2017jd026844 (2017).

8 Dunne, E. M. *et al.* Global atmospheric particle formation from CERN CLOUD measurements. *Science* **354**, 1119-1124, doi:10.1126/science.aaf2649 (2016).
